# Behavior Individuality: A Focus on *Drosophila melanogaster*

**DOI:** 10.3389/fphys.2021.719038

**Published:** 2021-11-30

**Authors:** Rubén Mollá-Albaladejo, Juan A. Sánchez-Alcañiz

**Affiliations:** Instituto de Neurociencias, UMH&CSIC, San Juan de Alicante, Spain

**Keywords:** behavior individuality, *Drosophila melanogaster*, animal personality, neurobiology, stochasticity

## Abstract

Among individuals, behavioral differences result from the well-known interplay of nature and nurture. Minute differences in the genetic code can lead to differential gene expression and function, dramatically affecting developmental processes and adult behavior. Environmental factors, epigenetic modifications, and gene expression and function are responsible for generating stochastic behaviors. In the last decade, the advent of high-throughput sequencing has facilitated studying the genetic basis of behavior and individuality. We can now study the genomes of multiple individuals and infer which genetic variations might be responsible for the observed behavior. In addition, the development of high-throughput behavioral paradigms, where multiple isogenic animals can be analyzed in various environmental conditions, has again facilitated the study of the influence of genetic and environmental variations in animal personality. Mainly, *Drosophila melanogaster* has been the focus of a great effort to understand how inter-individual behavioral differences emerge. The possibility of using large numbers of animals, isogenic populations, and the possibility of modifying neuronal function has made it an ideal model to search for the origins of individuality. In the present review, we will focus on the recent findings that try to shed light on the emergence of individuality with a particular interest in *D. melanogaster*.

## 1. Introduction

Individuality, temperament, behavioral syndromes, or animal personality are terms used to define the display of specific behavioral traits that are stable over time (Dall et al., 2004; Sih et al., 2004; Bell, 2007). At the population level, animals tend to show homogeneous behavior. However, if analyzed in more detail, it is clear that individuals within a group show behavioral patterns that differentiate them from the average. For example, in humans, food perception is highly personal, and it depends on the combination of both sociocultural experience and genetic polymorphisms that affect the function of gustatory (Kim et al., 2003) and olfactory receptors (Wysocki and Beauchamp, 1984; Kowalewski and Ray, 2020). This interindividual variation is not exclusive to humans and is generally observed in all living beings. For example, bacteria grown in the laboratory display variations in swimming behavior due to changes in gene expression, indicating that even in populations with the same genetic background and grown in similar conditions, heterogeneous behaviors can be observed (Davidson and Surette, 2008). Experiments with the clonal fish *Poecilia formosa* show that individuals grown in standardized conditions in isolation after birth display considerable differences in their behavior (Bierbach et al., 2017). Those results would suggest that stochastic developmental events lead to high variability in behavior. For example, variations in mushroom body size in *D. melanogaster*, affecting aggression, lifespan, and sleep, have been linked with polymorphisms in more than 100 genes (Zwarts et al., 2015). It is important to remark that those variations in behavior are consistent over time. We are not referring to just an acute change in their behavioral pattern or the minor variations resulting from the “noise” in the system that might induce temporary changes in a particular behavior (Faisal et al., 2008). Other stochastic events, inherent to any biological system, such as changes in gene expression or development, will have a more profound impact on the outcome of the behavior, contributing to persistent variations in behavior and the emergence of individuality (Honegger and de Bivort, 2018).

The genetic background of the organism and the developmental history of an individual dramatically affect how the animal will express this individuality (Dall et al., 2004; Sih et al., 2004; Wolf and Weissing, 2010). Although animals of the same species share the same genome, subtle changes during development (i.e., axon guidance) can have severe effects on the final connectivity of the neurons due to stochastic events altering specific behaviors (Linneweber et al., 2020; Kiral et al., 2021). In addition, environmental factors during growth and epigenetic changes will modify gene expression. It is important to remark that although animal personality defines the animal and shows specific stability, it has certain levels of plasticity, as happens with foraging behaviors on a day-to-day basis (Anreiter and Sokolowski, 2019). Previous experiences, growth and developmental conditions, and epigenetic factors form a complex milieu where behavior and individual differences emerge.

*D. melanogaster* is an outstanding model to study behavior individuality for several reasons. For example, it is possible to dispose of a large number of individuals to analyze per experiment; there is an extensive collection of isogenic lines available with sequenced genomes, and we can manipulate flies at the genetic level (Casillas and Barbadilla, 2017). Moreover, with only 100,000 neurons in the central brain (Raji and Potter, 2021) and a large collection of tools to manipulate neural circuits, *D. melanogaster* is an excellent model system to understand the genetic and neural basis of behavior heterogeneity.

The present review presents the scientific advances in the study of behavior individuality in *D. melanogaster*. We will cover the knowledge gained in genetics, development, epigenetics, and the methods used to study individual behavior in flies.

## 2. Genetic Basis of Animal Individuality

**Figure 1:**
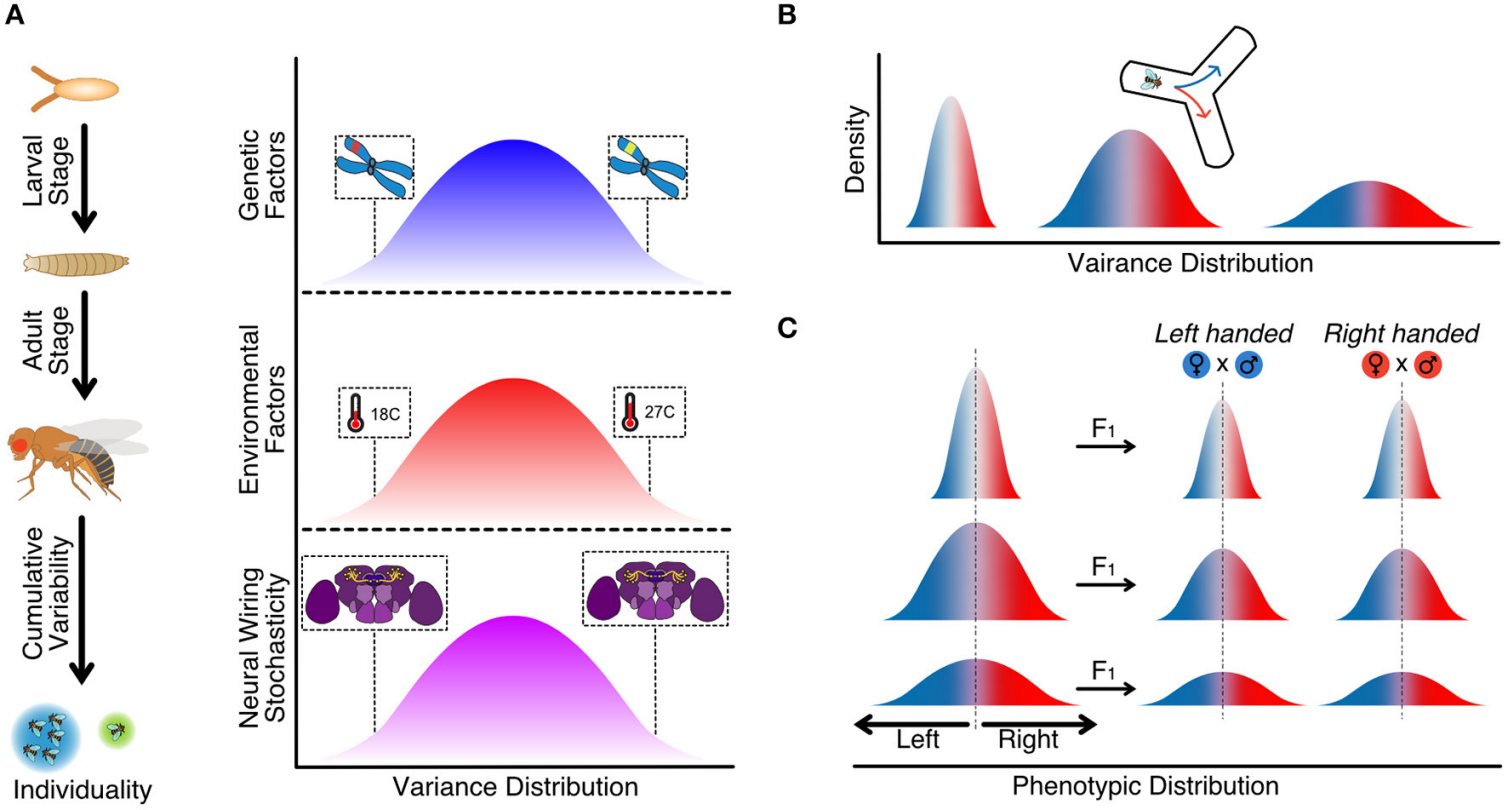
**(A)** Cumulative and relative contribution of different sources of interindividual variability from early development stages to adult life experience. Individuality emerges from the combination of genetic factors, environmental factors, and stochastic factors. **(B)** Variance distribution for a given phenotypic trait evoking variability among individuals within a population (Buchanan et al., 2015). **(C)** Variance distribution of phenotypic handedness is inherited to next generations within a population (Ayroles et al., 2015).

Animal behavior and, ultimately, animal personality results from the interaction of genetic and non-genetic factors. Indeed, animals combine genetic and environmental traits to promote specific adaptation to available resources from the environment and their gene expression adapts to the circumstances (Figure 1A) (Honegger and de Bivort, 2018; Koyama et al., 2020). However, neither nature nor nurture in isolation can explain how and why animals behave the way they do, as each of the two components has its weight. Even more, the expression of specific genes is not constant over time but can change through the life course of the animal, causing modifications in the personality of an individual (Juneja et al., 2016; Lin et al., 2016). Hence, genes are in constant communication with non-genetic factors coupling complex and sensitive networks that will finally define the personality of an individual in response to natural pressure.

The advent of genomics during the last decades has revolutionized the field of behavioral neuroscience, providing evidence on how genes can face natural dynamic variation between individuals and affect different behavioral outputs contributing to population performance (Jin et al., 2001; Ueno and Takahashi, 2020). How, when, and where a gene or genes are expressed and modulate brain activity and processing, could explain why two individuals from the same species, similar genetic backgrounds, and raised in similar conditions behave differently. For example, in *D. melanogaster* genetic variation in olfactory receptors (*Or22a/Or22b, Or35a, and Or47a*) affect different odor guidance perceptions among individuals of the same population (Richgels and Rollmann, 2011). Also, variation in odor guidance to 2,3-butanedione showed genes associated with neural development and the later processing in the central nervous system which might be related to that behavioral variation (Brown et al., 2013). This genotypic variation is seen not only at the behavioral level but also, for example, in lifespan or morphological and anatomical traits as brain, wing, thorax, or eye size (Carreira et al., 2016; Buchberger et al., 2021).

In behavioral neuroscience (and neuroscience in general), the use of inbred lines can reduce phenotypic variability. However, even in highly controlled experimental conditions, high levels of variability can be observed both in mice and *D. melanogaster* (Honegger and de Bivort, 2018). Many animals available and short reproductive time made it possible to generate numerous collections of isogenic lines. The Drosophila Genetic Reference Panel (DGRP) are a set of fly populations derived from subsequent inbred generations producing lines with sequenced genomes and all its polymorphisms annotated (Mackay et al., 2012). As the genome of each of those fly lines is sequenced, it is possible to test the variations in behavior for each line to later search for the genetic basis responsible for such behavior. Also, studies in simple stereotyped behaviors have found complex genetic architectures involved in the final output performance of specific behaviors that differ between individuals. Thirteen genes are involved in the flight performance in *D. melanogaster* where the variation in the expression and regulation of these genes may reflect the variation in the flight performance among individuals. Among them, polymorphisms at the regulatory region of the transmembrane tyrosine kinase, Egfr, showed the largest behavioral variation affecting wing shape (Spierer et al., 2021). Other studies have used DGRP lines to search genetic variation in aggressive behavior, virgin egg retention, or immune response against pathogens (i.e., *Coxiella burnetii*) (Akhund-Zade et al., 2017; Guzman et al., 2021). Interestingly, interindividual variation can also be observed in *D. melanogaster* mating behavior. During courtship, male flies execute a series of stereotyped and progressive behaviors that culminate in mating (Hall, 1994). Even in behavior as stereotyped as courtship, which must avoid interspecies mating, studies made in natural populations have observed variability in male courtship behaviors toward mated females (Ruedi and Hughes, 2008). Genome-Wide Association Studies (GWAS) performed in the inbred collection of DGRP flies showed the influence of genetic variation in courtship variability. Particularly, the transition from copulation to no engagement was associated with SNPs in *Serrate* and *Furin-1* genes (Gaertner et al., 2015).

A key question in behavior individuality is why animals show this range of erratic behaviors. A possible explanation derives from the natural variability in the surrounding environment. A genetically uniform population might be desirable in a stable environment with no threats, as individuals diverting from the average might not be well-adapted. However, in an ever-changing environment, animals must develop strategies to survive. One option would be phenotypic plasticity, where individuals have developed the ability to change their behavior upon environmental requirements. For example, fruit flies adapt their diet to environmental temperature. *D. melanogaster* feeds primarily from yeast, but if the temperature drops below 15°C, flies change to a plant-based diet. Plants provide the flies with unsaturated fatty acids, increasing cell membrane fluidity and lifespan (Brankatschk et al., 2018). Phenotypic plasticity can also be observed in overwintering *Drosophila*, winter morphs, where flies display different phenotypic plasticity to adapt to low temperatures (Panel et al., 2020; Stockton et al., 2020).

Another possibility is to hedge their options or diversified bet-hedging. In this scenario, a single genotype produces a distribution of phenotypes, assuring that at least some individuals within the population will be well-adapted to cope with any environmental change (Honegger and de Bivort, 2018). Under those circumstances, individuals show heterogeneous natural behavior. This natural variability would be heritable, causing the behavioral individuality observed. Experiments using wild-type and inbreed lines exploring the animal idiosyncrasies have studied the mechanistic behind animal handedness or better performance using left or right hand. This behavior can be observed in *D. melanogaster* when it is forced to choose to go left or right in an arena with no other stimulus. In this paradigm, flies showed considerable variability in this particular trait, which relates to specific genotypes (Ayroles et al., 2015; Buchanan et al., 2015). The authors showed that although each population averaged a 50% chance of turning either right or left, some were more variable, with more individuals either turning left or right (Figure 1B). Thus, the turning bias of individual flies was not heritable but was the degree of variability of the population. Furthermore, crossing two “righty” or two “lefty” individuals did not produce hybrids all “righty” and “lefty,” respectively, as the F1 progeny would show average turn vias of 50%. However, the variability of the particular line would be inherited (Figure 1C). A gene encoding an axon guidance molecule, *Tenascin-a*, has been proposed as a candidate involved in the observed behavior heterogeneity (refer to next section) (Ayroles et al., 2015; Buchanan et al., 2015). This distribution of phenotypes observed might be an evolutionary strategy, diversified bet-hedging, to guarantee that at least some individuals will be well-adapted when facing unpredictable environments. Bet-hedging could be the possible source of variation in the phototactic behavior of flies in two populations of flies from two different climates. Flies from very stable tropical regions where day/light time is relatively stable would show less variability in a phototactic choice assay than the ones from a nordic region, where seasonal changes in light/dark are more dramatic. Serotonin variation among the populations could be the source of such variation as feeding flies with serotonin would decrease variability (Kain et al., 2012; Krams et al., 2021). To reinforce this idea, other studies focused on other species such as *Caenorhabditis elegans* show that in isogenic sibling individuals raised under the same conditions, serotonin might regulate behavioral variability. Complete depletion of serotonin (*tph-1*) or some of the G-protein coupled receptors (SER-1, SER-4, and SER-7) induce changes in the individual roaming behavior across development (Stern et al., 2017).

As we have seen, some of those genes affect the development of specific neural circuits. In contrast, others affect neuromodulatory networks, such as serotonin in locomotor behavior in *C. elegans*. In other cases, mutations in particular alleles affect particular gene regulatory networks, ultimately affecting neuronal function. For example, it is the case of the chaperone *heat shock protein 90* (HSP90), involved in the folding and maturation of other proteins. HSP90 mutants show high levels of interindividual morphology variation (Rutherford and Lindquist, 1998). In addition, recent studies have shown that HSP90 mutant flies display a high interindividual variation in circadian motor control (Hung et al., 2009). Daily cycles of light and darkness can entrain the circadian clock. Once established, a gene network can keep it oscillating without environmental cues (Williams and Sehgal, 2001). While wild-type flies showed low variations in their rhythmic activity, flies with decreased activity of the chaperone HSP90 showed variation between individuals, from rhythmic and arrhythmic to other complex behaviors. Those results indicated that HSP90 could be acting as a capacitor of behavior individuality, affecting the degree of variation in circadian behavioral activity (Hung et al., 2009).

All these studies support the idea that in flies from the same population, there is an accumulation of polymorphisms due to spontaneous mutations, natural pressure, or simple genomic diversification from the average of the population, conferring different behavioral personalities among them. Consistent individual differences can result from intra-genotypic variations among individuals and differences in the value of state variables such as metabolic rate, growth rate, or energetic reserves (Amat et al., 2018). Also, stochastic gene expression may underlie the phenomenon of partial penetrance of mutations and variability that may interfere in individual personality (Topalidou and Chalfie, 2011).

## 3. Developmental and Growth Conditions Shape Animal Personality

In the previous section, we have discussed the genetic basis for behavioral variability. However, we also mentioned the critical role of the developmental process and growth in individual behaviors. We refer to the variations of behavior that are non-genetic as intragenotypic variation. This variability derives from stochastic microenvironmental effects such as temperature, isolation, or food sources that force individuals to adapt phenotypically to the environment (Becher et al., 2010).

Temperature is a wide-ranging environmental factor that flies can experience and must manage to maintain their homeostasis. In *D. melanogaster*, the life cycle takes longer at low temperatures and accumulates more fat energy stores as a mechanism to cope with possible future starvation periods (Klepsatel et al., 2019, 2020). Previous studies have shown that there is gene expression variation in response to low temperatures in *D. melanogaster* due to plasticity phenomena (Fry, 2008). Whole-genome sequencing in fly populations evolved in different temperatures has revealed the role of different genes in the recombination rate divergence between populations (Winbush and Singh, 2021). The transcription factors *chimo* and *eve* show different levels of expression between flies reared at different temperatures (25 vs. 17°C). This variation modifies the arborization of sensory neurons inducing interindividual variability perceiving temperature (Alpert et al., 2020; Huang et al., 2020). Those temperature changes affect synaptic connectivity in the *D. melanogaster* visual system, as flies grown at low temperatures (19°C) have more synapse numbers than the ones grown at higher temperatures (25°C) (Kiral et al., 2021). Furthermore, phenotypic plasticity in front of temperature variation is not exclusive of drosophilids as other social insects as honey bees show different learning abilities related to labors within the colony depending on the larvae developmental temperature (Tautz et al., 2003; Jeanson, 2019). Those results indicate that temperature is a major source of phenotypic plasticity and interindividual variability within a population.

Environmental factors can dramatically influence the development of the animal and condition its growth, modifying its behavior. Flies raised in stimulating naturalistic environment vials vs. vials without any enrichment that could match natural environments showed significant differences in fitness. Enriched populations showed higher intragenotypic variability for most of the behavioral traits measured, concluding that enrichment stimuli environment is one of the central sources of variability for behavior traits crucial to surviving (Akhund-Zade et al., 2019). Also, gene expression noise varies depending on the specific gene function, suggesting that variance in gene expression noise in order to evoke phenotypic plasticity may be beneficial for survival to environmental changes (Blake et al., 2006; Newman et al., 2006; Viney and Reece, 2013).

Even in conditions where genetic background and environment are kept constant, similar individuals can develop non-heritable idiosyncratic behaviors, morphology, and gene expression profiles evoking variability that could be consistent with development and life. Stochastic development wiring or minute differences in growth conditions can contribute to the trait under study. Identical populations of pea aphids and flies grown in identical environmental conditions display heterogeneous behaviors, eliminating the role of any internal factor (Schuett et al., 2011; Kain et al., 2012; Ayroles et al., 2015). Therefore, the role of those non-heritable traits in brain development and, therefore, in individual behavior is gaining importance in neuroscience. Studies carried on the visual orientation behavior in *D. melanogaster* showed that the Dorsal Cluster Neurons axonal projections within the medulla brain are a predictor of visual orientation, suggesting that stochastic variation in brain wiring evoke non-heritable behavioral variations (Linneweber et al., 2020). We mentioned in the previous section that different populations of flies showed variations in their handedness behavior. *Tenascin-a* encodes a cell surface protein involved in axon guidance and synaptogenesis. GWAS studies of the DGRP lines showed that this protein participates in the wiring of the neural circuits involved in locomotor behavior. Presumably, variations in the protein function might affect the synaptic connectivity of the neurons in the Central Complex of the fly brain, creating the high individual to individual variations, which ultimately will affect the apparent random choice, left or right, creating a bias in specific individuals (Ayroles et al., 2015; Buchanan et al., 2015). These studies indicate how intricate the relation between genes and environment is, showing that the genetic background of a population would determine the observed variability level, becoming heritable. However, the stochastic neuronal wiring in individuals can also be the source of particular behaviors.

These findings support the idea that stochastic variation in brain wiring and gene expression combined with different genetic traits are determinants of such behavior variability.

## 4. How Epigenetics Influence Behavior

Epigenetics involve any biological mechanisms that regulate the expression of genes without changing the DNA sequences, becoming the crossroad between the genetic and the environmental factors leading to a biological impact upon gene expression (Heard and Martienssen, 2014; Schuebel et al., 2016; Schiele and Domschke, 2018). Different factors influence epigenetic modifications such as diet, experience, characteristics of the ecosystem, lifestyle, and the physiological state of the individuals, impacting on disease outcome, social organization, and individual behavior, among others (Waterland and Jirtle, 2003; Cunliffe, 2016; Dawson et al., 2018; Baenas and Wagner, 2019).

Epigenetic modifications affect animals at the individual and social level, modifying the role of individuals inside specific social contexts (Anreiter et al., 2017; Sara et al., 2019). For example, honey bees display DNA methylation after intruders encounter, leading to aggressive behavior (Herb et al., 2018). Differential histone 3 (H3) acetylation (H3K27) affects morphologically and behaviorally *Camponotus floridanus* ant workers. Those modifications induce differences in foraging and scouting behaviors leading to high levels of task distribution (Simola et al., 2013, 2016; Yan et al., 2014). Epigenetic modifications also alter parasocial insects like the fruit fly behavioral, developmental, and physiological traits. For example, a low-protein diet induces H3K27 heterochromatin trimethylation shortening the lifespan of flies. In addition, acetylation of H3K27 by blocking the *Drosophila Polycomb* gene induces a dysregulation of the repression of homeotic genes (Tie et al., 2009, 2016). Furthermore, epigenetic regulation affects foraging behavior by histone methylation of the *for* (foraging) gene promoter *pr4* establishing a polymorphism between sitters and rovers behaviors in *D. melanogaster* (Anreiter et al., 2017). Other studies showed that euchromatin histone methyltransferase activity affects non-associative learning and courtship memory in *Drosophila* (Kramer et al., 2011).

## 5. Individuality in Collective Behavior

Animals coordinate their behavior with other individuals for benefits, including increased opportunities to mate, greater migratory and foraging efficiency, less chance of being attacked, and better energy costs (Handegard et al., 2012; Berdahl et al., 2013; Jolles et al., 2019). Several researchers have focused their research on the study of the neurogenetic bases of collective behavior in order to understand how individuals can form complex social networks among themselves, improving their survival as occurs in social animals like fishes, ducks, bees, or flies (Becher et al., 2010; Bialek et al., 2014; Ramdya et al., 2017).

Behavior individuality within a colony can emerge from self-organization and social interactions benefiting host hospitalization and decision-making processes (Jeanson, 2019). Individual roles within an animal social network can change over time as an evolutionary method to decrease disease transmission (Stroeymeyt et al., 2018). In other cases, biological roles can be persistent for each individual, such as birds taking turns as alarm-calling sentinels in the colony or the task distribution in ant colonies, respectively (Nagy et al., 2010; Yan et al., 2014; Ramdya et al., 2017; Friedman et al., 2020). Individuals in social networks experience social encounters to spread information from informed to uninformed to transmit beneficial information for survival and relevant future decision-making processes (Canright and Engø-Monsen, 2006). Different studies revealed that fruit flies coordinate their oviposition sites based on the information shared by experienced flies through social encounters. Those experiments suggest that highly clustered flies show a high potential to spread information among individuals (Pasquaretta et al., 2016). Besides, flies are aware of the number of individuals and adjust their interactive behavior to the group size (Rooke et al., 2020). Other studies have shown that collective aggregation depends on external stimuli. For example, the *Poxn* transcription factor and Orco co-receptor are involved in the chemical detection of fly cuticle hydrocarbon pheromones that may be involved in clustering mechanisms (Schneider et al., 2012b). In addition, the mechanoreceptor NompC is involved in collective behavior as *NompC* mutant flies only avoid noxious CO_2_ when are clustered with wild-type flies compared with isolated *NompC* mutant ones. These results indicate that there is spread of information from wild type flies to *NompC* mutants (Ramdya et al., 2015). These studies, in addition to others, support the idea that fruit flies integrate sensory information in order to drive appropriate collective behavior and facilitate social learning and foraging decisions in larvae and adulthood to buffer efficiently environmental stress (Tinette et al., 2004; Billen, 2006; Lihoreau et al., 2016; Dombrovski et al., 2017; Jolles et al., 2017; Jiang et al., 2020).

Despite all the benefits derived from the establishment of social networks within a group of individuals, *D. melanogaster* has a parasocial organization where collective and individual behavior remains cohesive. Each group member behaving differently could explain that the cascade of group motion likely emerges from specific individual patterns of behavior (Rosenthal et al., 2015). Nevertheless, there is no evidence that some individuals act as leaders beginning the clustering within groups of fruit flies. However, the aggregation process grows as more flies join the pioneer ones, affecting information spreading (Jiang et al., 2020).

Even if *D. melanogaster* organization does not fit in a eusocial pattern where there is specific and hierarchical task distribution, the presence of individual behavior heterogeneity may drive crucial collective behavior beneficial for both the individual and the conspecifics individuals. Thus, understanding the dynamics of collective behavior in the fruit fly may guide understanding the neurogenetic bases involved and how the behavioral patterns of animal societies arise.

## 6. Methods to Study Behavior Individuality

**Figure 2:**
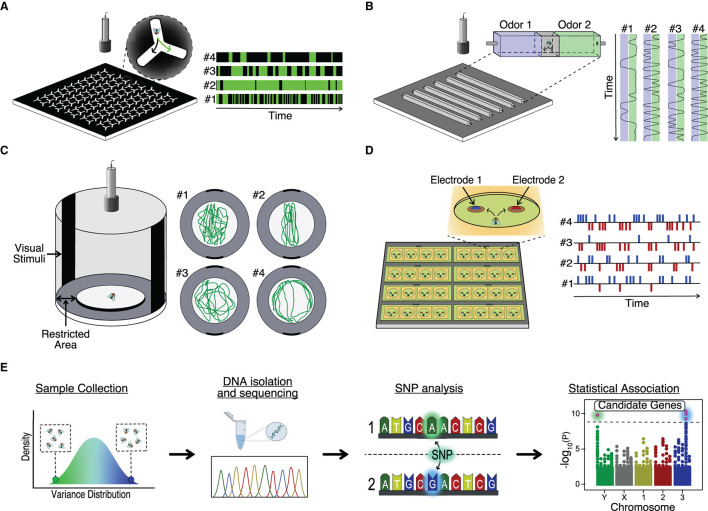
High throughput analysis of behavior variance. **(A)** Schematic set up for study locomotor handedness variability by Y-maze (Ayroles et al., 2015; Buchanan et al., 2015). **(B)** Schematic setup for study olfactory guidance variability. Adapted from Honegger et al. (2019). **(C)** Schematic Buridian arena to study visual orientation variability (Colomb et al., 2012). **(D)** Schematic FlyPAD set-up to study feeding microstructure variability (Itskov et al., 2014). **(E)** Genome-Wide Association Studies (GWAS) workflow.

*D. melanogaster* has emerged as an excellent model to study behavior individuality for several reasons: the small size and short breeding time allow us to obtain large quantities of animals to test in small setups in short periods of time; there is a large number of inbred lines with sequenced and annotated genomes to search for the possible genetic basis of individuality, and; finally, the possibility to manipulate the genome and neurons of the flies allow us to test ultimately how the candidate genes (and neural circuits) affect the generation of behavioral variation (Venken et al., 2011). The number of behavioral paradigms developed to study *Drosophila* (and in general animal behavior) have blossomed in the last years due to an increase in computer capacity and the development of machine learning algorithms dedicated to it. In Figure 2, we describe some hardware (with custom associated software) used to study behavior in individual flies. For example, the classic Y-maze, where flies can choose between two paths, is scaled up to allow multiple simultaneous recordings of individual flies. With this high-throughput system, the behavior of 25,000 individuals was analyzed and permitted the study of the neural and genetic basis of handedness in flies, identifying candidate genes and neurons (Buchanan et al., 2015) (Figure 2A). As mentioned in previous sections, animals, and particularly *D. melanogaster* display idiosyncratic behavioral responses to odors. To study this behavior, Honegger et al. build a paradigm arena where individual flies could choose between two odors emanating from opposing ends of a corridor. By video tracking the fly behavior, it was possible to show how neuromodulation was involved in the preferential choice of individuals (Figure 2B) (Honegger et al., 2019). Other innate behaviors like object orientation responses can be analyzed in a high-throughput manner using multiple Buridian paradigm arenas and video tracking (Figure 2C). Using this set up, the authors demonstrated that stochastic developmental events were altering the Dorsal Cluster Neuron circuits of different individuals, leading to idiosyncratic behaviors in flies (Linneweber et al., 2020). Finally, it is also possible to study feeding in *D. melanogaster*. *FlyPad* is an automated high-throughput method to study fly ingestion in individual flies. This system would allow the analysis of behavior individuality in, for example, the ability of flies to choose between two types of foods (Itskov et al., 2014) (Figure 2D). Recently, an upgrade in *FlyPad* named *OptoPad* allows optogenetically modifying the activity of selected circuits in real-time by ectopically expressing channelrhodopsins in those neurons. With this method, it is possible to couple the feeding activity of the fly with the modification of the neural activity in a closed-loop manner (Moreira et al., 2019).

The previously described methods are focused on the analysis of individual flies. However, the social context is lost, and although flies are non-eusocial insects, flies can aggregate both *in vitro* (Jiang et al., 2020) and in the wild (Soto-Yéber et al., 2019). The formation of *Drosophila* clusters is motivated by the presence of mating partners or food, with pheromones like cis-Vaccenyl acetate (Bartelt et al., 1985) and neuromodulators like serotonin (Sun et al., 2020) playing an essential role. As shown in the previous section, individuality can affect collective behavior. For example, within a group, some individuals display “bold” or “shy” behaviors. Newer software, like *idtracker.ai*, can identify each individual unequivocally in large groups (i.e., up to 70 flies), track overtime their trajectory, and the interaction with other members of the group (Romero-Ferrero et al., 2019). Finally, it is essential to mention that all those methods require standardized procedures, which start with the breeding conditions. Different mediums to homogenize the growth of flies can decrease variability due to external conditions (Piper et al., 2014). Table 1 contains a thorough description of many hardware and software developed in recent years that are applied or can be applied to study behavior individuality. Most of the software and hardware listed are open source and shared with any laboratory that requests its use. We indicate the species for which they were designed or most used. However, researchers can modify the published versions to adapt them to their model system of interest. We apologize in advance for the methods we forgot to mention or did not find, as many appear constantly.

**Table 1:** Overview of automated and high throughput software and hardware for animal behavior analysis.

**Hardware/software**	**Utility**	**Software/ hardware**	**Programming language**	Species^*^	**References**
AnTrax	Tracking software for color-tagged individuals of small species	Software	Matlab	*Ooceraea biroi*	Gal et al., 2020
Automated *Drosophila* Olfactory Conditioning System	Automated software and hardware system to study olfactory behavior coupled with learning and memory assessment	Software and Hardware	Arduino and Labview	*Drosophila melanogaster*	Jiang et al., 2016
BEEtag	Image tracking software to track labeled identified individual bees or anatomical markers	Software	Matlab	*Apis mellifera*	Crall et al., 2015
Buritrack	Tracking software either in the presence or in the absence of visual targets in a Buridian paradigm setup	Software and Hardware	R	Different species	Colomb et al., 2012
ClockLab	Analysis of circadian locomotor activity data collected using DAM system	Software	Matlab	*Drosophila melanogaster*	Pfeiffenberger et al., 2010
CTrax	Tracking software for automatically quantify individual and social behavior of fruit flies	Software	Matlab	*Drosophila melanogaster*	Branson et al., 2009
DAM	*Drosophila Activity Monitor*. System from Trikinetics for locomotion, sleep and circadian rhythms activity quantification	Hardware	None	*Drosophila melanogaster*	www.trikinetics.com
DART	*Drosophila Arousal Tracking*. Hardware and software that reports locomotor and positional activity data of individual flies in multiple chambers	Software and Hardware	Matlab	*Drosophila melanogaster*	Faville et al., 2015
DeepLabCut	Markerless pose estimation based on machine learning with deep neural networks that achieves excellent results with minimal training data to study behavior by tracking various body parts	Software	Python	*Mus musculus* and *Drosophila melanogaster*	Mathis et al., 2018
DeepPoseKit	Machine learning software for deep estimation of pose location to analyze specific behavior parameters	Software	Python	Different species	Graving et al., 2019
DIAS	*Dynamic Image Analysis System*. Tracking software to analyze locomotor behavior in the adult fruit fly as in other individuals	Software	Matlab	*Drosophila melanogaster*	Slawson et al., 2009
*Drosophila* Island	Algorithm that quantify locomotor and flight activity behavior from fruit flies on specific Island platforms	Software	Fiji and R	*Drosophila melanogaster*	Eidhof et al., 2017
Ethoscopes	Machine learning software to track and profile behavior in real time while trigger stimulus to flies in a feedback-loop mode	Software	R	*Drosophila melanogaster*	Geissmann et al., 2017
Expresso	Automated feeding hardware to measure individual meal-bouts with high temporal and volume resolution	Hardware	Matlab	*Drosophila melanogaster*	Yapici et al., 2016
FIM / FIMTrack	*FTIR-based Imaging Method*. Tracking hardware and software to study locomotion behavior based on internal reflection of infrared light (FTIR) operating at all wavelengths allowing *in vivo* detection of fluorescent proteins	Software and Hardware	C++	*Drosophila melanogaster*	Risse et al., 2013
FLIC	*Fly Liquid-Food Interaction Counter*. Automated hardware to detect and quantify physical contact with liquid food to study feeding behavior in fruit flies	Software and Hardware	Matlab	*Drosophila melanogaster*	Ro et al., 2014
Flyception	Retroreflective based tracking coupled with imaging brain activity on free walking fruit flies	Hardware	C++	*Drosophila melanogaster*	Grover et al., 2020
FlyGrAM	*Fly Group Activity Monitor*. Software for monitoring real-time group locomotion based on background subtraction	Software	Python	*Drosophila melanogaster*	Scaplen et al., 2019
FlyMAD	*Fly Mind-Altering Device*. Infrared laser targeting hardware for accurately thermogenetic silencing or activation on freely walking flies	Hardware	None	*Drosophila melanogaster*	Bath et al., 2014
FlyPAD	*Fly Proboscis and Activity Detector*. Detailed, automated and high-throughput quantification of feeding behavior based on capacitance data	Software and Hardware	Matlab	*Drosophila melanogaster*	Itskov et al., 2014
FlyPEZ	High-throughput hardware system to rapidly analyze individual fly behavior with tracking and controlled sensory or optogenetic stimulation	Hardware	Matlab	*Drosophila melanogaster*	Williamson et al., 2018
Flywalk	Automatic olfactory preference tracking hardware for screening individual flies	Hardware	Matlab	*Drosophila melanogaster*	Steck et al., 2012
Idtrackerai	Individual tracking of all trajectories from small and large collectives with high identification accuracy	Software	Python	Different species	Romero-Ferrero et al., 2019
Imaging system for zebrafish larvae behavior analyses	Three-camera imaging system hardware to image zebrafish larvae behavior in front of visual stimuli provided by specific slides in a high-throughput manner	Hardware	None	*Danio rerio*	Richendrfer and Créton, 2013
JAABA	Machine learning-based system for automatically quantify different animal behavior parameters	Software	Matlab	Different species	Kabra et al., 2013
Machine learning tracking software	Machine learning-based tracking software for individual trajectories inside a group	Software	None	Insects	Wario et al., 2017
pySOLO	Sleep and locomotor activity software analyzer of multiple isolated flies	Software	Python	*Drosophila melanogaster*	Gilestro, 2012
RFID	Radiofrequency identification based tracking hardware on individual ID infrared detection by antennas	Hardware	Matlab	Different species	Schneider et al., 2012a; Torquet et al., 2018; Reinert et al., 2019
RING	*Rapid Iterative Negative Geotaxis*. Digital photography based hardware to measure negative geotaxis in individual or collective animal groups simultaneously	Hardware	Scion Image - Pascal	*Drosophila melanogaster*	Gargano et al., 2005
The Tracked Program	Tracking of small movements at any location on a DAM set up to study sleep behavior and structure	Software	Java	*Drosophila melanogaster*	Donelson et al., 2012
WormFarm	Integrated microfluidic hardware to quantify different behaviors such as survival from images and videos	Hardware	None	*Caenorhabditis elegans*	Xian et al., 2013

* *Species for which the hardware or software was initially designed. Nevertheless, most of them can be adapted to other species*.

After describing the behavior of interest, we need to understand such behavior's genetic and neural basis. The advancement of genomic tools like Next Generation Sequencing, QTL, and GWAS is helping to understand how genes relate to behavioral traits (Bengston et al., 2018) (Figure 1E). Furthermore, we have seen the advantage of working with already sequenced collections of isogenic lines (Mackay et al., 2012). In addition, now we can do experiments to study large natural populations of flies and sequence all individuals with excellent coverage depth at low cost.

Finally, the study of the neural circuitry involved in behavior, *D. melanogaster*, is an excellent system as many different transgenic lines have been created to label, trace, and analyze complete neural circuits (Jenett et al., 2012). Similar to *C. elegans*, we aim to map all synaptic partners in the brain of *D. melanogaster* through electron microscopy (EM). Although we still need to map the whole brain, the connectome for the central brain already exists (Scheffer et al., 2020). So far, those data provide a standalone image of the *Drosophila* brain. However, it would be desirable to have a full EM reconstruction of each individual's brain analyzed, although this looks right now as an impossible effort. Even for smaller organisms, it is a daunting task, but it could be beneficial as a recent work where EM reconstruction of eight *C. elegans* brains showed variations in synaptic connectivity between them, making each brain unique (Witvliet et al., 2021). As we learn more about the circuitry involved in particular behaviors, it might be possible in the future to focus our EM reconstruction efforts on small regions of the brain known to control particular behaviors. We could then reconstruct those regions synaptically for multiple individuals, gaining excellent knowledge regarding neural circuit variability between individuals.

## 7. Conclusions

How do heritable (genetic) and non-heritable (stochastic events) factors interact to shape behavior? It could be possible that individuals carrying particular polymorphisms might be more susceptible to environmental changes, leading to enough variations among individuals to show specific individual persistent behaviors. It would not be easy to differentiate between the genetic and non-genetic basis of such behavior as there is a constant interplay in this particular case. To add more complexity to the problem epigenetic modifications, alter gene function. It means we cannot just focus our efforts on finding particular genetic sequences as the final goal, as we need to understand how genomes change along with the life of an individual. Finally, from a behavioral point of view, we are constantly talking about individuality. At the same time, animals modify their behaviors during their lifetime as they interact with other conspecifics. All this information indicates that the emergence of individuality or animal personality requires the study at different levels.

The latest advancements and development of high-throughput sequencing have finally opened the door to looking for the genetic basis of animal individuality and how the environment affects gene expression. We know individual animals show particular personalities, from flies to mice, monkeys to humans. However, at this very moment, we can start thinking to move from pure ethological studies to the molecular dissection of those behaviors. Neural circuitry tracing and reconstruction through electron microscopy are helping to build a map of the neural connections of the brain. So far, we do not have more than a few individuals. However, understanding and dissecting those circuits might help us finally understand how the expression of particular genes during a particular period or the subtle variations in connectivity could lead to a deeper understanding of individuality.

It is intriguing that nervous systems, like many other biological systems, are plastic within certain boundaries, so we can expect that personal individuality will be expressed deferentially over time or under certain environmental circumstances. *D. melanogaster* offers an excellent model system as we can test our hypothesis in large groups of animals in a short period of time (Buchanan et al., 2015). In addition, we can control to a large degree the genetic variation of our population by using inbred lines (Ayroles et al., 2015; Linneweber et al., 2020). The generation of the DGRP lines has helped advance this field, as controlling the genetic variation of the populations of interest can help us narrow down the candidate genetic variants, if any, or discard the genetic variation and ascribe it to stochastic developmental processes.

We have focused on the genetic and epigenetic changes that alter individual behavior. We have also studied how stochastic developmental processes alter neural connectivity leading to interindividual variation. However, another possible source of potential behavioral variability might come from the interaction of individuals with environmental microbes, from *Wolbachia* infections to changes in the gut microbiome. In this particular case, no genetic variation or neural circuit alteration would be responsible for the change in behavior. It is known that *Wolbachia* infection affects different *D. melanogaster* behaviors such as sleep (Bi et al., 2018), temperature preference (Truitt et al., 2019), or aggression (Rohrscheib et al., 2015). Alteration in the gut microbiome can affect aggression in *Drosophila* males (Jia et al., 2021) or sleep and memory (Silva et al., 2021). Those results point to the interaction of individuals with microorganisms as another potential source of interindividual behavior variability that must be taken into consideration.

Finally, from an evolutionary point of view, individuality might play an essential role in providing an adaptative advantage. For example, we have described that animals might use diversified bet-hedging as a mechanism to produce high levels of variation within a population to ensure that at least some individuals will be well-adapted when facing unpredictable environments. Although more experimental evidence accumulates to support this theory, without any doubt, we are in front of a growing field of knowledge that will evolve soon.

## Author Contributions

RM-A and JS-A conceived and wrote the manuscript. All authors contributed to the article and approved the submitted version.

## Funding

This study was supported by grants from the Generalitat Valenciana, CIDEGENT program (CIDEGENT/2018/035), Spanish Ministry of Science and Innovation (PID2019-105839GA-I00) and Program Ramón y Cajal (RyC2019-026747-I).

## Conflict of Interest

The authors declare that the research was conducted in the absence of any commercial or financial relationships that could be construed as a potential conflict of interest.

## Publisher's Note

All claims expressed in this article are solely those of the authors and do not necessarily represent those of their affiliated organizations, or those of the publisher, the editors and the reviewers. Any product that may be evaluated in this article, or claim that may be made by its manufacturer, is not guaranteed or endorsed by the publisher.

## References

[B1] Akhund-Zade J. Bergland A. O. Crowe S. O. Unckless R. L. (2017). The genetic basis of natural variation in Drosophila (Diptera: Drosophilidae) virgin egg retention. J. Insect Sci. 17. 10.1093/jisesa/iew094 28042107 PMC5270406

[B2] Akhund-Zade J. Ho S. O'Leary C. de Bivort B. (2019). The effect of environmental enrichment on behavioral variability depends on genotype, behavior, and type of enrichment. J. Exp. Biol. 222. 10.1242/jeb.202234 31413102

[B3] Alpert M. H. Frank D. D. Kaspi E. Flourakis M. Zaharieva E. E. Allada R.. (2020). A circuit encoding absolute cold temperature in Drosophila. Curr. Biol. 30, 2275.e5–2288.e5. 10.1016/j.cub.2020.04.038 32442464 PMC7314653

[B4] Amat I. Desouhant E. Gomes E. Moreau J. Monceau K. (2018). Insect personality: what can we learn from metamorphosis? Curr. Opin. Insect Sci. 27, 46–51. 10.1016/j.cois.2018.02.014 30025634

[B5] Anreiter I. Kramer J. M. Sokolowski M. B. (2017). Epigenetic mechanisms modulate differences in Drosophila foraging behavior. Proc. Natl. Acad. Sci. U.S.A. 114, 12518–12523. 10.1073/pnas.1710770114 29078350 PMC5703298

[B6] Anreiter I. Sokolowski M. B. (2019). The foraging gene and its behavioral effects: pleiotropy and plasticity. Annu. Rev. Genet. 53, 373–392. 10.1146/annurev-genet-112618-043536 31487469

[B7] Ayroles J. F. Buchanan S. M. O'Leary C. Skutt-Kakaria K. Grenier J. K. Clark A. G.. (2015). Behavioral idiosyncrasy reveals genetic control of phenotypic variability. Proc. Natl. Acad. Sci. U.S.A. 112:6706. 10.1073/pnas.1503830112 25953335 PMC4450409

[B8] Baenas N. Wagner A. E. (2019). *Drosophila melanogaster* as an alternative model organism in nutrigenomics. Genes Nutr. 14:14. 10.1186/s12263-019-0641-y 31080523 PMC6501408

[B9] Bartelt R. J. Schaner A. M. Jackson L. L. (1985). Cis-vaccenyl acetate as an aggregation pheromone inDrosophila *melanogaster*. J. Chem. Ecol. 11, 1747–1756. 10.1007/BF01012124 24311338

[B10] Bath D. E. Stowers J. R. Hörmann D. Poehlmann A. Dickson B. J. Straw A. D. (2014). FlyMAD: Rapid thermogenetic control of neuronal activity in freely walking Drosophila. Nat. Methods 11, 756–762. 10.1038/nmeth.2973 24859752

[B11] Becher M. A. Hildenbrandt H. Hemelrijk C. K. Moritz R. F. (2010). Brood temperature, task division and colony survival in honeybees: a model. Ecol. Modell. 221, 769–776. 10.1016/j.ecolmodel.2009.11.016

[B12] Bell A. M. (2007). Future directions in behavioural syndromes research. Proc. R. Soc. B Biol. Sci. 274, 755–761. 10.1098/rspb.2006.0199 17251088 PMC1919401

[B13] Bengston S. E. Dahan R. A. Donaldson Z. Phelps S. M. van Oers K. Sih A.. (2018). Genomic tools for behavioural ecologists to understand repeatable individual differences in behaviour. Nat. Ecol. Evol. 2, 944–955. 10.1038/s41559-017-0411-4 29434349 PMC9437744

[B14] Berdahl A. Torney C. J. Ioannou C. C. Faria J. J. Couzin I. D. (2013). Emergent sensing of complex environments by mobile animal groups. Science 339, 574–576. 10.1126/science.1225883 23372013

[B15] Bi J. Sehgal A. Williams J. A. Wang Y.-F. (2018). Wolbachia affects sleep behavior in *Drosophila melanogaster*. J. Insect Physiol. 107, 81–88. 10.1016/j.jinsphys.2018.02.011 29499213

[B16] Bialek W. Cavagna A. Giardina I. Mora T. Pohl O. Silvestri E.. (2014). Social interactions dominate speed control in poising natural flocks near criticality. Proc. Natl. Acad. Sci. U.S.A. 111, 7212–7217. 10.1073/pnas.1324045111 24785504 PMC4034227

[B17] Bierbach D. Laskowski K. L. Wolf M. (2017). Behavioural individuality in clonal fish arises despite near-identical rearing conditions. Nat. Commun. 8:15361. 10.1038/ncomms15361 28513582 PMC5442312

[B18] Billen J. (2006). Signal variety and communication in social insects. Proc. Neth. Entomol. Soc. Meet 17, 9–25.

[B19] Blake W. J. Balázsi G. Kohanski M. A. Isaacs F. J. Murphy K. F. Kuang Y.. (2006). Phenotypic consequences of promoter-mediated transcriptional noise. Mol. Cell 24, 853–865. 10.1016/j.molcel.2006.11.003 17189188

[B20] Brankatschk M. Gutmann T. Knittelfelder O. Palladini A. Prince E. Grzybek M.. (2018). A temperature-dependent switch in feeding preference improves Drosophila development and survival in the cold. Dev. Cell 46, 781.e4–793.e4. 10.1016/j.devcel.2018.05.028 30253170

[B21] Branson K. Robie A. A. Bender J. Perona P. Dickinson M. H. (2009). High-throughput ethomics in large groups of Drosophila. Nat. Methods 6, 451–457. 10.1038/nmeth.1328 19412169 PMC2734963

[B22] Brown E. B. Layne J. E. Zhu C. Jegga A. G. Rollmann S. M. (2013). Genome-wide association mapping of natural variation in odour-guided behaviour in Drosophila. Genes Brain Behav. 12, 503–515. 10.1111/gbb.12048 23682951

[B23] Buchanan S. M. Kain J. S. de Bivort B. L. (2015). Neuronal control of locomotor handedness in Drosophila. Proc. Natl. Acad. Sci. U.S.A. 112:6700. 10.1073/pnas.1500804112 25953337 PMC4450378

[B24] Buchberger E. Bilen A. Ayaz S. Salamanca D. Matas de las Heras C. Niksic A.. (2021). Variation in pleiotropic hub gene expression is associated with interspecific differences in head shape and eye size in Drosophila. Mol. Biol. Evol. 38, 1924–1942. 10.1093/molbev/msaa335 33386848 PMC8097299

[B25] Canright G. S. Engø-Monsen K. (2006). Spreading on networks: a topographic view. Complexus 3, 131–146. 10.1159/000094195

[B26] Carreira V. P. Mensch J. Hasson E. Fanara J. J. (2016). Natural genetic variation and candidate genes for morphological traits in *Drosophila melanogaster*. PLoS ONE 11:e0160069. 10.1371/journal.pone.0160069 27459710 PMC4961385

[B27] Casillas S. Barbadilla A. (2017). Molecular population genetics. Genetics 205, 1003–1035. 10.1534/genetics.116.196493 28270526 PMC5340319

[B28] Colomb J. Reiter L. Blaszkiewicz J. Wessnitzer J. Brembs B. (2012). Open source tracking and analysis of adult Drosophila locomotion in Buridan's paradigm with and without visual targets. PLoS ONE 7:e42247. 10.1371/journal.pone.0042247 22912692 PMC3415391

[B29] Crall J. D. Gravish N. Mountcastle A. M. Combes S. A. (2015). BEEtag: a low-cost, image-based tracking system for the study of animal behavior and locomotion. PLoS ONE 10:e0136487. 10.1371/journal.pone.0136487 26332211 PMC4558030

[B30] Cunliffe V. T. (2016). The epigenetic impacts of social stress: how does social adversity become biologically embedded? Epigenomics 8, 1653–1669. 10.2217/epi-2016-0075 27869483 PMC5289034

[B31] Dall S. R. X. Houston A. I. McNamara J. M. (2004). The behavioural ecology of personality: consistent individual differences from an adaptive perspective. Ecol. Lett. 7, 734–739. 10.1111/j.1461-0248.2004.00618.x28794486

[B32] Davidson C. J. Surette M. G. (2008). Individuality in bacteria. Annu. Rev. Genet. 42, 253–268. 10.1146/annurev.genet.42.110807.091601 18652543

[B33] Dawson E. H. Bailly T. P. M. Dos Santos J. Moreno C. Devilliers M. Maroni B.. (2018). Social environment mediates cancer progression in Drosophila. Nat. Commun. 9:3574. 10.1038/s41467-018-05737-w 30177703 PMC6120865

[B34] Dombrovski M. Poussard L. Moalem K. Kmecova L. Hogan N. Schott E.. (2017). Cooperative behavior emerges among Drosophila larvae. Curr. Biol. 27, 2821.e2–2826.e2. 10.1016/j.cub.2017.07.054 28918946

[B35] Donelson N. Kim E. Z. Slawson J. B. Vecsey C. G. Huber R. Griffith L. C. (2012). High-resolution positional tracking for long-term analysis of Drosophila sleep and locomotion using the “tracker?? program. PLoS ONE 7:e37250. 10.1371/journal.pone.0037250 22615954 PMC3352887

[B36] Eidhof I. Fenckova M. Elurbe D. M. van de Warrenburg B. Nobau A. C. Schenck A. (2017). High-throughput analysis of locomotor behavior in the Drosophila island assay. J. Visual. Exp. 2017, 1–11. 10.3791/55892 29155762 PMC5755321

[B37] Faisal A. A. Selen L. P. J. Wolpert D. M. (2008). Noise in the nervous system. Nat. Rev. Neurosci. 9, 292–303. 10.1038/nrn2258 18319728 PMC2631351

[B38] Faville R. Kottler B. Goodhill G. J. Shaw P. J. Van Swinderen B. (2015). How deeply does your mutant sleep? Probing arousal to better understand sleep defects in Drosophila. Sci. Rep. 5:8454. 10.1038/srep08454 25677943 PMC4326961

[B39] Friedman D. Johnson B. Linksvayer T. (2020). Distributed physiology and the molecular basis of social life in eusocial insects. Hormones Behav. 122:104757. 10.1016/j.yhbeh.2020.104757 32305342

[B40] Fry J. D. (2008). Genotype-environment interaction for total fitness in Drosophila. J. Genet. 87:355. 10.1007/s12041-008-0058-7 19147925

[B41] Gaertner B. E. Ruedi E. A. McCoy L. J. Moore J. M. Wolfner M. F. Mackay T. F. C. (2015). Heritable variation in courtship patterns in *Drosophila melanogaster*. Genes Genomes Genet. 5, 531–539. 10.1534/g3.114.014811 25650358 PMC4390569

[B42] Gal A. Saragosti J. Kronauer D. J. (2020). antrax, a software package for high-throughput video tracking of color-tagged insects. eLife 9:e58145. 10.7554/eLife.58145 33211008 PMC7676868

[B43] Gargano J. W. Martin I. Bhandari P. Grotewiel M. S. (2005). Rapid iterative negative geotaxis (RING): a new method for assessing age-related locomotor decline in Drosophila. Exp. Gerontol. 40, 386–395. 10.1016/j.exger.2005.02.005 15919590

[B44] Geissmann Q. Garcia Rodriguez L. Beckwith E. J. French A. S. Jamasb A. R. Gilestro G. F. (2017). Ethoscopes: an open platform for high-throughput ethomics. PLoS Biol. 15:e2003026. 10.1371/journal.pbio.2003026 29049280 PMC5648103

[B45] Gilestro G. F. (2012). Video tracking and analysis of sleep in *Drosophila melanogaster*. Nat. Protoc. 7, 995–1007. 10.1038/nprot.2012.041 22538850

[B46] Graving J. M. Chae D. Naik H. Li L. Koger B. Costelloe B. R.. (2019). Deepposekit, a software toolkit for fast and robust animal pose estimation using deep learning. eLife 8, 1–42. 10.7554/eLife.47994 31570119 PMC6897514

[B47] Grover D. Katsuki T. Li J. Dawkins T. J. Greenspan R. J. (2020). Imaging brain activity during complex social behaviors in Drosophila with flyception2. Nat. Commun. 11:623. 10.1038/s41467-020-14487-7 32001689 PMC6992788

[B48] Guzman R. M. Howard Z. P. Liu Z. Oliveira R. D. Massa A. T. Omsland A.. (2021). Natural genetic variation in *Drosophila melanogaster* reveals genes associated with *Coxiella burnetii* infection. Genetics 217. 10.1093/genetics/iyab005 33789347 PMC8045698

[B49] Hall J. C. (1994). The mating of a fly. Science 264, 1702–1714. 10.1126/science.8209251 8209251

[B50] Handegard N. O. Boswell K. M. Ioannou C. C. Leblanc S. P. Tjøstheim D. B. Couzin I. D. (2012). The dynamics of coordinated group hunting and collective information transfer among schooling prey. Curr. Biol. 22, 1213–1217. 10.1016/j.cub.2012.04.050 22683262

[B51] Heard E. Martienssen R. A. (2014). Transgenerational epigenetic inheritance: myths and mechanisms. Cell 157, 95–109. 10.1016/j.cell.2014.02.045 24679529 PMC4020004

[B52] Herb B. R. Shook M. S. Fields C. J. Robinson G. E. (2018). Defense against territorial intrusion is associated with DNA methylation changes in the honey bee brain. BMC Genomics 19:216. 10.1186/s12864-018-4594-0 29580210 PMC5870497

[B53] Honegger K. de Bivort B. (2018). Stochasticity, individuality and behavior. Curr. Biol. 28, R8-R12. 10.1016/j.cub.2017.11.058 29316423

[B54] Honegger K. S. Smith M. A.-Y. Churgin M. A. Turner G. C. de Bivort B. L. (2019). Idiosyncratic neural coding and neuromodulation of olfactory individuality in Drosophila. Proc. Natl. Acad. Sci. U.S.A. 10.1073/pnas.1901623116 31455738 PMC7519279

[B55] Huang W. Carbone M. A. Lyman R. F. Anholt R. R. H. Mackay T. F. C. (2020). Genotype by environment interaction for gene expression in *Drosophila melanogaster*. Nat. Commun. 11:5451. 10.1038/s41467-020-19131-y 33116142 PMC7595129

[B56] Hung H.-C. Kay S. A. Weber F. (2009). Hsp90, a capacitor of behavioral variation. J. Biol. Rhythms 24, 183–192. 10.1177/0748730409333171 19465695

[B57] Itskov P. M. Moreira J.-M. Vinnik E. Lopes G. Safarik S. Dickinson M. H.. (2014). Automated monitoring and quantitative analysis of feeding behaviour in Drosophila. Nat. Commun. 5:4560. 10.1038/ncomms5560 25087594 PMC4143931

[B58] Jeanson R. (2019). Within-individual behavioural variability and division of labour in social insects. J. Exp. Biol. 222. 10.1242/jeb.190868 31127006

[B59] Jenett A. Rubin G. M. Ngo T.-T. Shepherd D. Murphy C. Dionne H.. (2012). A gal4-driver line resource for Drosophila neurobiology. Cell Rep. 2, 991–1001. 10.1016/j.celrep.2012.09.011 23063364 PMC3515021

[B60] Jia Y. Jin S. Hu K. Geng L. Han C. Kang R.. (2021). Gut microbiome modulates Drosophila aggression through octopamine signaling. Nat. Commun. 12:2698. 10.1038/s41467-021-23041-y 33976215 PMC8113466

[B61] Jiang H. Hanna E. Gatto C. L. Page T. L. Bhuva B. Broadie K. (2016). A fully automated Drosophila olfactory classical conditioning and testing system for behavioral learning and memory assessment. J. Neurosci. Methods 261, 62–74. 10.1016/j.jneumeth.2015.11.030 26703418 PMC4749449

[B62] Jiang L. Cheng Y. Gao S. Zhong Y. Ma C. Wang T.. (2020). Emergence of social cluster by collective pairwise encounters in *Drosophila*. eLife 9:e51921. 10.7554/eLife.51921 31959283 PMC6989122

[B63] Jin W. Riley R. M. Wolfinger R. D. White K. P. Passador-Gurgell G. Gibson G. (2001). The contributions of sex, genotype and age to transcriptional variance in *Drosophila melanogaster*. Nat. Genet. 29, 389–395. 10.1038/ng766 11726925

[B64] Jolles J. W. Boogert N. J. Sridhar V. H. Couzin I. D. Manica A. (2017). Consistent individual differences drive collective behavior and group functioning of schooling fish. Curr. Biol. 27, 2862.e7–2868.e7. 10.1016/j.cub.2017.08.004 28889975 PMC5628957

[B65] Jolles J. W. King A. J. Killen S. S. (2019). The role of individual heterogeneity in collective animal behaviour. Trends Ecol. Evol. 10.1016/j.tree.2019.11.001 31879039

[B66] Juneja P. Quinn A. Jiggins F. M. (2016). Latitudinal clines in gene expression and cis-regulatory element variation in *Drosophila melanogaster*. BMC Genomics 17:981. 10.1186/s12864-016-3333-7 27894253 PMC5126864

[B67] Kabra M. Robie A. A. Rivera-Alba M. Branson S. Branson K. (2013). Jaaba: interactive machine learning for automatic annotation of animal behavior. Nat. Methods 10, 64–67. 10.1038/nmeth.2281 23202433

[B68] Kain J. S. Stokes C. de Bivort B. L. (2012). Phototactic personality in fruit flies and its suppression by serotonin and white. Proc. Natl. Acad. Sci. U.S.A. 109, 19834–19839. 10.1073/pnas.1211988109 23150588 PMC3511718

[B69] Kim U.-K. Jorgenson E. Coon H. Leppert M. Risch N. Drayna D. (2003). Positional cloning of the human quantitative trait locus underlying taste sensitivity to phenylthiocarbamide. Science 299, 1221–1225. 10.1126/science.1080190 12595690

[B70] Kiral F. R. Dutta S. B. Linneweber G. A. Poppa C. von Kleist M. Hassan B. A.. (2021). Variable brain wiring through scalable and relative synapse formation in Drosophila. bioRxiv. 10.1101/2021.05.12.443860

[B71] Klepsatel P. Girish T. N. Gáliková M. (2020). Acclimation temperature affects thermal reaction norms for energy reserves in Drosophila. Sci. Rep. 10:21681. 10.1038/s41598-020-78726-z 33303846 PMC7729904

[B72] Klepsatel P. Wildridge D. Gáliková M. (2019). Temperature induces changes in Drosophila energy stores. Sci. Rep. 9:5239. 10.1038/s41598-019-41754-5 30918312 PMC6437209

[B73] Kowalewski J. Ray A. (2020). Predicting human olfactory perception from activities of odorant receptors. iScience 23. 10.1016/j.isci.2020.101361 32731170 PMC7393469

[B74] Koyama T. Texada M. J. Halberg K. A. Rewitz K. (2020). Metabolism and growth adaptation to environmental conditions in Drosophila. Cell. Mol. Life Sci. 77, 4523–4551. 10.1007/s00018-020-03547-2 32448994 PMC7599194

[B75] Kramer J. M. Kochinke K. Oortveld M. A. W. Marks H. Kramer D. de Jong E. K.. (2011). Epigenetic regulation of learning and memory by Drosophila EHMT/G9a. PLoS Biol. 9:e1000569. 10.1371/journal.pbio.1000569 21245904 PMC3014924

[B76] Krams I. A. Krama T. Krams R. Trakimas G. Popovs S. Jøers P.. (2021). Serotoninergic modulation of phototactic variability underpins a bet-hedging strategy in *Drosophila melanogaster*. Front. Behav. Neurosci. 15:66. 10.3389/fnbeh.2021.659331 33935664 PMC8085305

[B77] Lihoreau M. Clarke I. M. Buhl J. Sumpter D. J. T. Simpson S. J. (2016). Collective selection of food patches in Drosophila. J. Exp. Biol. 219, 668–675. 10.1242/jeb.127431 26747899

[B78] Lin Y. Chen Z. X. Oliver B. Harbison S. T. (2016). Microenvironmental gene expression plasticity among individual *Drosophila melanogaster*. Genes Genomes Genetics 6, 4197–4210. 10.1534/g3.116.035444 27770026 PMC5144987

[B79] Linneweber G. A. Andriatsilavo M. Dutta S. B. Bengochea M. Hellbruegge L. Liu G.. (2020). A neurodevelopmental origin of behavioral individuality in the Drosophila visual system. Science 367, 1112–1119. 10.1126/science.aaw7182 32139539

[B80] Mackay T. F. C. Richards S. Stone E. A. Barbadilla A. Ayroles J. F. Zhu D.. (2012). The *Drosophila melanogaster* genetic reference panel. Nature 482, 173–178. 10.1038/nature10811 22318601 PMC3683990

[B81] Mathis A. Mamidanna P. Cury K. M. Abe T. Murthy V. N. Mathis M. W.. (2018). DeepLabCut: markerless pose estimation of user-defined body parts with deep learning. Nat. Neurosci. 21, 1281–1289. 10.1038/s41593-018-0209-y 30127430

[B82] Moreira J.-M. Itskov P. M. Goldschmidt D. Baltazar C. Steck K. Tastekin I.. (2019). optopad, a closed-loop optogenetics system to study the circuit basis of feeding behaviors. eLife 8:e43924. 10.7554/eLife.43924 31226244 PMC6589098

[B83] Nagy M. Ákos Z. Biro D. Vicsek T. (2010). Hierarchical group dynamics in pigeon flocks. Nature 464, 890–893. 10.1038/nature08891 20376149

[B84] Newman J. R. S. Ghaemmaghami S. Ihmels J. Breslow D. K. Noble M. DeRisi J. L.. (2006). Single-cell proteomic analysis of *S. cerevisiae* reveals the architecture of biological noise. Nature 441, 840–846. 10.1038/nature04785 16699522

[B85] Panel A. D. C. Pen I. Pannebakker B. A. Helsen H. H. M. Wertheim B. (2020). Seasonal morphotypes of Drosophila suzukii differ in key life-history traits during and after a prolonged period of cold exposure. Ecol. Evol. 10, 9085–9099. 10.1002/ece3.6517 32953048 PMC7487234

[B86] Pasquaretta C. Battesti M. Klenschi E. Bousquet C. A. H. Sueur C. Mery F. (2016). How social network structure affects decision-making in *Drosophila melanogaster*. Proc. R. Soc. B Biol. Sci. 283:20152954. 10.1098/rspb.2015.2954 26936247 PMC4810861

[B87] Pfeiffenberger C. Lear B. C. Keegan K. P. Allada R. (2010). Processing circadian data collected from the Drosophila activity monitoring (DAM) system. Cold Spring Harbor Protoc. 2010:5519. 10.1101/pdb.prot5519 21041392

[B88] Piper M. D. W. Blanc E. Leitão-Gonçalves R. Yang M. He X. Linford N. J.. (2014). A holidic medium for *Drosophila melanogaster*. Nat. Methods 11, 100–105. 10.1038/nmeth.2731 24240321 PMC3877687

[B89] Raji J. I. Potter C. J. (2021). The number of neurons in Drosophila and mosquito brains. PLoS ONE 16:e0250381. 10.1371/journal.pone.0250381 33989293 PMC8121336

[B90] Ramdya P. Lichocki P. Cruchet S. Frisch L. Tse W. Floreano D.. (2015). Mechanosensory interactions drive collective behaviour in Drosophila. Nature 519, 233–236. 10.1038/nature14024 25533959 PMC4359906

[B91] Ramdya P. Schneider J. Levine J. D. (2017). The neurogenetics of group behavior in *Drosophila melanogaster*. J. Exp. Biol. 220, 35–41. 10.1242/jeb.141457 28057826

[B92] Reinert J. K. Schaefer A. T. Kuner T. (2019). High-throughput automated olfactory phenotyping of group-housed mice. Front. Behav. Neurosci. 13:267. 10.3389/fnbeh.2019.00267 31920577 PMC6927946

[B93] Richendrfer H. Créton R. (2013). Automated high-throughput behavioral analyses in zebrafish larvae. J. Visual. Exp. 7, 1–6. 10.3791/50622 23851916 PMC3731428

[B94] Richgels P. K. Rollmann S. M. (2011). Genetic variation in odorant receptors contributes to variation in olfactory behavior in a natural population of *Drosophila melanogaster*. Chem. Senses 37, 229–240. 10.1093/chemse/bjr097 22038943 PMC3278676

[B95] Risse B. Thomas S. Otto N. Löpmeier T. Valkov D. Jiang X.. (2013). FIM, a novel FTIR-based imaging method for high throughput locomotion analysis. PLoS ONE 8:e53963. 10.1371/journal.pone.0053963 23349775 PMC3549958

[B96] Ro J. Harvanek Z. M. Pletcher S. D. (2014). FLIC: high-throughput, continuous analysis of feeding behaviors in Drosophila. PLoS ONE 9:e101107. 10.1371/journal.pone.0101107 24978054 PMC4076220

[B97] Rohrscheib C. E. Bondy E. Josh P. Riegler M. Eyles D. van Swinderen B.. (2015). Wolbachia influences the production of octopamine and affects Drosophila male aggression. Appl. Environ. Microbiol. 81, 4573–4580. 10.1128/AEM.00573-15 25934616 PMC4551182

[B98] Romero-Ferrero F. Bergomi M. G. Hinz R. C. Heras F. J. H. de Polavieja G. G. (2019). idtracker.ai: tracking all individuals in small or large collectives of unmarked animals. Nat. Methods 16, 179–182. 10.1038/s41592-018-0295-5 30643215

[B99] Rooke R. Rasool A. Schneider J. Levine J. D. (2020). *Drosophila melanogaster* behaviour changes in different social environments based on group size and density. Commun. Biol. 3:304. 10.1038/s42003-020-1024-z 32533063 PMC7293324

[B100] Rosenthal S. B. Twomey C. R. Hartnett A. T. Wu H. S. Couzin I. D. (2015). Revealing the hidden networks of interaction in mobile animal groups allows prediction of complex behavioral contagion. Proc. Natl. Acad. Sci. U.S.A. 112, 4690–4695. 10.1073/pnas.1420068112 25825752 PMC4403201

[B101] Ruedi E. A. Hughes K. A. (2008). Natural genetic variation in complex mating behaviors of male *Drosophila melanogaster*. Behav. Genet. 38, 424–436. 10.1007/s10519-008-9204-5 18369720

[B102] Rutherford S. L. Lindquist S. (1998). Hsp90 as a capacitor for morphological evolution. Nature 396, 336–342. 10.1038/24550 9845070

[B103] Sara M. Merrill N. G. Kobor M. S. (2019). Social environment and epigenetics. Curr. Top. Behav. Neurosci. 42, 289–320. 10.1007/7854_2019_114 31485989

[B104] Scaplen K. M. Mei N. J. Bounds H. A. Song S. L. Azanchi R. Kaun K. R. (2019). Automated real-time quantification of group locomotor activity in *Drosophila melanogaster*. Sci. Rep. 9, 1–16. 10.1038/s41598-019-40952-5 30872709 PMC6418093

[B105] Scheffer L. K. Xu C. S. Januszewski M. Lu Z. Takemura S.-,y. Hayworth K. J.. (2020). A connectome and analysis of the adult *Drosophila* central brain. eLife 9:e57443. 10.7554/eLife.57443 32880371 PMC7546738

[B106] Schiele M. A. Domschke K. (2018). Epigenetics at the crossroads between genes, environment and resilience in anxiety disorders. Genes Brain Behav. 17:e12423. 10.1111/gbb.12423 28873274

[B107] Schneider C. W. Tautz J. Grünewald B. Fuchs S. (2012a). RFID tracking of sublethal effects of two neonicotinoid insecticides on the foraging behavior of *Apis mellifera*. PLoS ONE 7:e30023. 10.1371/journal.pone.0030023 22253863 PMC3256199

[B108] Schneider J. Dickinson M. H. Levine J. D. (2012b). Social structures depend on innate determinants and chemosensory processing in Drosophila. Proc. Natl. Acad. Sci. U.S.A. 109(Suppl 2), 17174–17179. 10.1073/pnas.1121252109 22802679 PMC3477376

[B109] Schuebel K. Gitik M. Domschke K. Goldman D. (2016). Making sense of epigenetics. Int. J. Neuropsychopharmacol. 19:pyw058. 10.1093/ijnp/pyw058 27312741 PMC5137275

[B110] Schuett W. Dall S. R. X. Baeumer J. Kloesener M. H. Nakagawa S. Beinlich F.. (2011). Personality variation in a clonal insect: the pea aphid, acyrthosiphon pisum. Dev. Psychobiol. 53, 631–640. 10.1002/dev.20538 21365642

[B111] Sih A. Bell A. M. Johnson J. C. Ziemba R. E. (2004). Behavioral syndromes: an integrative overview. Q. Rev. Biol. 79, 241–277. 10.1086/422893 15529965

[B112] Silva V. Palacios-Munoz A. Okray Z. Adair K. L. Waddell S. Douglas A. E.. (2021). The impact of the gut microbiome on memory and sleep in Drosophila. J. Exp. Biol. 224:jeb233619. 10.1242/jeb.233619 33376141 PMC7875489

[B113] Simola D. F. Graham R. J. Brady C. M. Enzmann B. L. Desplan C. Ray A.. (2016). Epigenetic (re)programming of caste-specific behavior in the ant camponotus floridanus. Science 351:6268. 10.1126/science.aac6633 26722000 PMC5057185

[B114] Simola D. F. Wissler L. Donahue G. Waterhouse R. M. Helmkampf M. Roux J.. (2013). Social insect genomes exhibit dramatic evolution in gene composition and regulation while preserving regulatory features linked to sociality. Genome Res. 23, 1235–1247. 10.1101/gr.155408.113 23636946 PMC3730098

[B115] Slawson J. B. Kim E. Z. Griffith L. C. (2009). High-resolution video tracking of locomotion in adult *Drosophila melanogaster*. J. Visual. Exp. 24, 1–3. 10.3791/1096 19390509 PMC2762895

[B116] Soto-Yéber L. Soto-Ortiz J. Godoy P. Godoy-Herrera R. (2019). The behavior of adult Drosophila in the wild. PLoS ONE 13:e0209917. 10.1371/journal.pone.0209917 30596767 PMC6312304

[B117] Spierer A. N. Mossman J. A. Smith S. P. Crawford L. Ramachandran S. Rand D. M. (2021). Natural variation in the regulation of neurodevelopmental genes modifies flight performance in Drosophila. PLoS Genet. 17:e1008887. 10.1371/journal.pgen.1008887 33735180 PMC7971549

[B118] Steck K. Veit D. Grandy R. Badia S. B. I. Mathews Z. Verschure P.. (2012). A high-throughput behavioral paradigm for Drosophila olfaction - the Flywalk. Sci. Rep. 2, 1–9. 10.1038/srep00361 22511996 PMC3328172

[B119] Stern S. Kirst C. Bargmann C. I. (2017). Neuromodulatory control of long-term behavioral patterns and individuality across development. Cell 171, 1649.e10–1662.e10. 10.1016/j.cell.2017.10.041 29198526

[B120] Stockton D. G. Wallingford A. K. Brind'amore G. Diepenbrock L. Burrack H. Leach H.. (2020). Seasonal polyphenism of spotted-wing Drosophila is affected by variation in local abiotic conditions within its invaded range, likely influencing survival and regional population dynamics. Ecol. Evol. 10, 7669–7685. 10.1002/ece3.6491 32760556 PMC7391339

[B121] Stroeymeyt N. Grasse A. V. Crespi A. Mersch D. P. Cremer S. Keller L. (2018). Social network plasticity decreases disease transmission in a eusocial insect. Science 362, 941–945. 10.1126/science.aat4793 30467168

[B122] Sun Y. Qiu R. Li X. Cheng Y. Gao S. Kong F.. (2020). Social attraction in Drosophila is regulated by the mushroom body and serotonergic system. Nat. Commun. 11:5350. 10.1038/s41467-020-19102-3 33093442 PMC7582864

[B123] Tautz J. Maier S. Groh C. Rössler W. Brockmann A. (2003). Behavioral performance in adult honey bees is influenced by the temperature experienced during their pupal development. Proc. Natl. Acad. Sci. U.S.A. 100, 7343–7347. 10.1073/pnas.1232346100 12764227 PMC165877

[B124] Tie F. Banerjee R. Fu C. Stratton C. A. Fang M. Harte P. J. (2016). Polycomb inhibits histone acetylation by CBP by binding directly to its catalytic domain. Proc. Natl. Acad. Sci. U.S.A. 113, E744-E753. 10.1073/pnas.1515465113 26802126 PMC4760827

[B125] Tie F. Banerjee R. Stratton C. A. Prasad-Sinha J. Stepanik V. Zlobin A.. (2009). Cbp-mediated acetylation of histone h3 lysine 27 antagonizes Drosophila polycomb silencing. Development 136, 3131–3141. 10.1242/dev.037127 19700617 PMC2730368

[B126] Tinette S. Zhang L. Robichon A. (2004). Cooperation between Drosophila flies in searching behavior. Genes Brain Behav. 3, 39–50. 10.1046/j.1601-183x.2003.0046.x 14960014

[B127] Topalidou I. Chalfie M. (2011). Shared gene expression in distinct neurons expressing common selector genes. Proc. Natl. Acad. Sci. U.S.A. 108, 19258–19263. 10.1073/pnas.1111684108 22087002 PMC3228421

[B128] Torquet N. Marti F. Campart C. Tolu S. Nguyen C. Oberto V.. (2018). Social interactions impact on the dopaminergic system and drive individuality. Nat. Commun. 9:3081. 10.1038/s41467-018-05526-5 30082725 PMC6079008

[B129] Truitt A. M. Kapun M. Kaur R. Miller W. J. (2019). Wolbachia modifies thermal preference in *Drosophila melanogaster*. Environ. Microbiol. 21, 3259–3268. 10.1111/1462-2920.14347 29971900 PMC6766989

[B130] Ueno T. Takahashi Y. (2020). Intrapopulation genetic variation in the level and rhythm of daily activity in Drosophila immigrans. Ecol. Evol. 10, 14388–14393. 10.1002/ece3.7041 33391722 PMC7771174

[B131] Venken K. J. T. Simpson J. H. Bellen H. J. (2011). Genetic manipulation of genes and cells in the nervous system of the fruit fly. Neuron 72, 202–230. 10.1016/j.neuron.2011.09.021 22017985 PMC3232021

[B132] Viney M. Reece S. E. (2013). Adaptive noise. Proc. R. Soc. B Biol. Sci. 280:20131104. 10.1098/rspb.2013.1104 23902900 PMC3735249

[B133] Wario F. Wild B. Rojas R. Landgraf T. (2017). Automatic detection and decoding of honey bee waggle dances. PLoS ONE 12:e0188626. 10.1371/journal.pone.0188626 29236712 PMC5728493

[B134] Waterland R. A. Jirtle R. L. (2003). Transposable elements: targets for early nutritional effects on epigenetic gene regulation. Mol. Cell. Biol. 23, 5293–5300. 10.1128/MCB.23.15.5293-5300.2003 12861015 PMC165709

[B135] Williams J. A. Sehgal A. (2001). Molecular components of the circadian system in Drosophila. Annu. Rev. Physiol. 63, 729–755. 10.1146/annurev.physiol.63.1.729 11181974

[B136] Williamson W. R. Peek M. Y. Breads P. Coop B. Card G. M. (2018). Tools for rapid high-resolution behavioral phenotyping of automatically isolated Drosophila. Cell Rep. 25, 1636.e5–1649.e5. 10.1016/j.celrep.2018.10.048 30404015

[B137] Winbush A. Singh N. D. (2021). Genomics of recombination rate variation in temperature-evolved *Drosophila melanogaster* Populations. Genome Biol. Evol. 13, 1–18. 10.1093/gbe/evaa252 33247719 PMC7851596

[B138] Witvliet D. Mulcahy B. Mitchell J. K. Meirovitch Y. Berger D. R. Wu Y.. (2021). Connectomes across development reveal principles of brain maturation. Nature. 10.1038/s41586-021-03778-8 34349261 PMC8756380

[B139] Wolf M. Weissing F. J. (2010). An explanatory framework for adaptive personality differences. Philos. Trans. R. Soc. B Biol. Sci. 365, 3959–3968. 10.1098/rstb.2010.0215 21078648 PMC2992748

[B140] Wysocki C. J. Beauchamp G. K. (1984). Ability to smell androstenone is genetically determined. Proc. Natl. Acad. Sci. U.S.A. 81, 4899–4902. 10.1073/pnas.81.15.4899 6589634 PMC391599

[B141] Xian B. Shen J. Chen W. Sun N. Qiao N. Jiang D.. (2013). WormFarm: A quantitative control and measurement device toward automated *Caenorhabditis elegans* aging analysis. Aging Cell 12, 398–409. 10.1111/acel.12063 23442149

[B142] Yan H. Simola D. F. Bonasio R. Liebig J. Berger S. L. Reinberg D. (2014). Eusocial insects as emerging models for behavioural epigenetics. Nat. Rev. Genet. 15, 677–688. 10.1038/nrg3787 25200663

[B143] Yapici N. Cohn R. Schusterreiter C. Ruta V. Vosshall L. B. (2016). A taste circuit that regulates ingestion by integrating food and hunger signals. Cell 165, 715–729. 10.1016/j.cell.2016.02.061 27040496 PMC5544016

[B144] Zwarts L. Vanden Broeck L. Cappuyns E. Ayroles J. F. Magwire M. M. Vulsteke V.. (2015). The genetic basis of natural variation in mushroom body size in *Drosophila melanogaster*. Nat. Commun. 6:10115. 10.1038/ncomms10115 26656654 PMC4682101

